# The Counterbalancing Role of Oxygen Vacancy between the Electrochromic Properties and the Trapping Effect Passivation for Amorphous Tungsten Oxide Films

**DOI:** 10.1002/smsc.202300219

**Published:** 2024-01-20

**Authors:** Zhaocheng Zhang, Huajing Mo, Ruicong Li, Xinglong Zhou, Zicong Lin, Jiong Zhang, Xiufeng Tang, Yunfeng Zhan, Jianyi Luo

**Affiliations:** ^1^ School of Applied Physics and Materials Wuyi University Jiangmen 529020 China; ^2^ School of Civil Engineering and Architecture Wuyi University Jiangmen 529020 China; ^3^ Research Center of Flexible Sensing Materials and Device Application Technology Wuyi University Jiangmen 529020 China

**Keywords:** amorphous tungsten oxides, electrochromics, oxygen vacancies the trapping effects

## Abstract

Defect engineering of electrode materials is considered highly effective in regulating their performance, among which oxygen vacancies play a vital role. Thereupon, comprehensively understanding effects of oxygen vacancy in electrochemical processes of transition metal oxides stays hot and controversial, representatively for amorphous tungsten oxide films and their electrochromic (EC) behaviors. Upon long‐term cycling, amorphous tungsten oxide suffers from the universal trapping effect governed by the intrinsic host microstructure and transport kinetics of the inserted ions, implying that manipulating oxygen vacancies could be a potential solution to the ion‐trapping problem. Hence, systematic work is urgent for not only tackling the trapping effect but also understanding the effect of oxygen vacancies on EC behaviors. Herein, the concentration of oxygen vacancies in the amorphous tungsten oxide films is modulated over a wide range. In combination with comprehensive experiments and first‐principles calculations, the presence of oxygen vacancy is detrimental to the EC properties, but it greatly attenuates the trapping effect. Excellent cyclic stability is achieved with a 100% optical modulation rate and charge capacity retention after 5000 cyclic voltammetry cycles. This study elucidates understanding of oxygen vacancy engineering in transition metal oxides, particularly regarding trapping effect passivation.

## Graphical abstract

Oxygen vacancy plays the counterbalancing role between the electrochromic properties and the trapping effect passivation for amorphous tungsten oxide films in their electrochromic behavior.

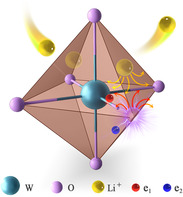

## 1 Introduction

Transition metal oxides are state‐of‐the‐art electrode materials in intercalation chemistry^[^ ^1^ ^]^ and when modified by defect engineering, they exhibit significantly different behaviors upon chemical and electrochemical reactions.^[^ ^2^, ^3^ ^]^ As an important and immensely interesting form of defect engineering, oxygen vacancy has been the focus of investigations in many disciplines.^[^ ^4^, ^5^, ^6^ ^]^ Representatively in electrochemistry, oxygen vacancy tuning led to the acceleration of the rate of surface incorporation reactions in CeO_2−*σ*_^[^ ^7^ ^]^ electrochemical properties of molybdenum oxide films were greatly modified for different applications,^[^ ^8^ ^]^ a new‐type and prominent electrochromism was achieved.^[^ ^9^ ^]^ Therefore, tuning of material properties by deliberate deviations in the oxygen occupancy from ideal stoichiometry lies at the heart of oxide chemistry.

Among the electrochemical properties, electrochromism can facilitate energy storage, color modulation, and solar radiation regulation under a small direct current (DC) voltage and has attracted much attention for decades.^[^ ^10^, ^11^ ^]^ Tungsten oxide, especially in its amorphous form, is one of the most intensively investigated electrochromic (EC) materials featuring high optical contrast and superior stability against ultraviolet radiation for outdoor applications.^[^ ^12^ ^]^ Efforts have been made to customize the properties of tungsten oxide, through stacking/compounding,^[^ ^13^, ^14^ ^]^ crystallization, and microstructure regulation,^[^ ^15^, ^16^, ^17^ ^]^ enabling its usage in a wide range of applications, including in today's smart windows,^[^ ^18^, ^19^ ^]^ newly emerging all‐solid‐state and self‐coloring EC technology,^[^ ^20^, ^21^ ^]^ lithium‐ion batteries,^[^ ^22^ ^]^ and supercapacitors.^[^ ^23^ ^]^ However, amorphous tungsten oxide suffers from ion‐trapping‐induced degradation of charge capacity, optical modulation span, and structural integrity upon prolonged electrochemical cycling,^[^ ^24^, ^25^, ^26^ ^]^ namely bad cyclic stability. This “trapping effect” was mainly attributed to the intrinsic structure–property. Juan Bisquert proposed that there were two types of ion trap sites in the WO_3_ host structure including “shallow ions sites,” enabling the diffusion of intercalated ions throughout the film, and “deep ions sites,” trapping the inserted charge for longer times.^[^ ^27^ ^]^ Further evidence was presented for three kinds of traps that degraded EC tungsten oxide during ion intercalation: 1) shallow traps eroding the colored state; 2) deep traps lowering the bleached‐state transmittance; and 3) irreversible traps.^[^ ^28^, ^29^ ^]^ Recently, to intrinsically probe the dynamic degradation evolution of WO_3_ films, the mass change,^[^ ^30^ ^]^ hysteretic ions transport kinetics,^[^ ^31^ ^]^ and mechanical stress/strain induced by insertion/extraction of ions^[^ ^32^ ^]^ were monitored. Thus, the internal mechanism of the “trapping effect” has been thoroughly studied but no effective solutions were proposed.

Considering the “trapping effect” to be mainly governed by the intrinsic microstructure of host WO_3,_ and transport kinetics of the inserted ions, regulating oxygen vacancy could be a novel potential solution for the ion‐trapping bottleneck because oxygen vacancy is located in the smallest crystal unit and greatly influences the aforementioned factors.^[^ ^33^, ^34^ ^]^ However, the effects of oxygen vacancies on the EC behavior of amorphous WO_3_ films have been disputed. Lee et al. first demonstrated that there exists a concentration threshold for oxygen vacancies to render EC character to amorphous WO_3−*y*_, and the coloration efficiency increased with increasing oxygen vacancies.^[^ ^35^ ^]^ This behavior was mainly attributed to the improved film conductivity, similar to that reported for crystalline WO_3−*y*_.^[^ ^36^ ^]^ Conversely, Niklasson and co‐workers pointed out that the presence of oxygen vacancies would introduce W^5+^—+^5+^ bonding and attenuate the optical regulating capability,^[^ ^37^ ^]^ which was concurred in the recent work by Dasgupta et al. on EC molybdenum oxide.^[^ ^38^ ^]^ On the other hand, He and Chiu's research revealed that there existed an optimal oxygen vacancy concentration for transmission control applications,^[^ ^39^ ^]^ and such a conclusion was also verified in NiO_*x*_^[^ ^40^ ^]^ and V_2_O_5−*x*_.^[^ ^41^ ^]^ This controversy is understandable because the EC process involves optical, electrical, electronic, electrochemical, structural, and mass‐transport kinetic properties. Hence, it is challenging to obtain a comprehensive understanding of the multiple roles of oxygen vacancy in the EC behavior of WO_3_ electrodes. Further, it is important to decode the influences of oxygen vacancies on “the trapping effect” and cyclic stability with an in‐depth analysis of underlying mechanisms. Addressing these challenges is paramount, not only for supplementing defect engineering and science in the EC field but also for solving the trapping effect and electrode failure problem for further investigations and applications of transition metal oxides.

This study attempts to provide comprehensive insight into the complicated effects of oxygen vacancies on the EC behavior of amorphous WO_3−*y*_ films. Modulation of oxygen vacancies in WO_3−*y*_ films over a wide range was achieved by tuning the Ar/O_2_ flow rate during film deposition by reactive sputtering. The evolution of the optical, electrical, electronic, and EC properties and the cyclic stability of the WO_3−*y*_ films were systematically recorded, and the feasibility of passivating “the trapping effect” through oxygen vacancy regulation was discussed.

## 2 Results and Discussion

**Figure 1:**
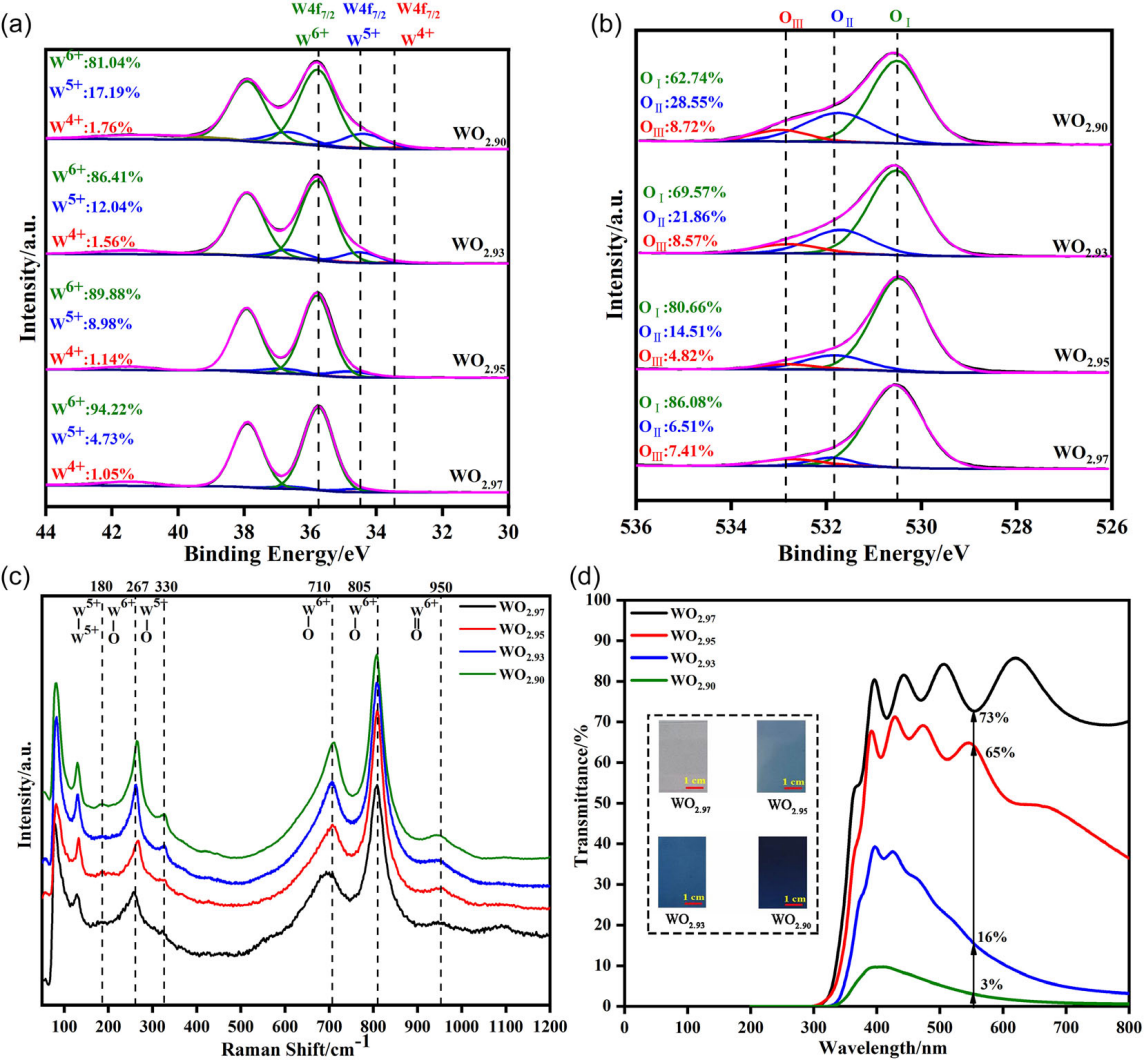
Characterization of tungsten oxide films deposited at different O_2_/Ar flow ratios. a) XPS images of W4*f*. b) XPS images of O1*s*. c) Raman spectra. d) Transmittance spectra and original‐state digital photographs are shown as insets.

During the DC reactive magnetron sputtering, the atmospheric components inside the sputtering chamber dominate the basic characteristics of the deposited tungsten oxide films. The absence of diffraction peaks in the XRD spectra (illustrated in Figure S2, Supporting Information) showed that all the as‐deposited tungsten oxide films were amorphous. All films were found to be composed of agglomerated nanoparticles without obvious differences in the surface morphology and appeared compact (corresponding SEM images shown in Figure S1 and S3, Supporting Information), suggesting that the Ar/O_2_ flow rate had little effect on the microstructure and crystallinity of the as‐deposited tungsten oxide films. Thus, the potential influences of the morphology and crystallinity on the research results were eliminated. **Figure** **1**a,b shows the W4*f* and O1*s* XPS core‐level spectra. The W4*f* spectra consisted of one pair of doublets at 35.6 ± 0.1 eV for W^6+^4*f*_7/2_, 34.3 ± 0.2 eV for W^5+^4*f*_7/2_, and 33.2 ± 0.2 eV for W^4+^4*f*_7/2_.^[^ ^42^ ^]^ Through integral‐area analysis of these three spectra, relative contents of W^6+^, W^5+^, and W^4+^ were calculated and shown as insets on the left side of each spectra. It can be seen that with an increase in the Ar/O_2_ flow rate from 40 sccm:3 sccm to 16 sccm:6 sccm, little or no W^4+^ was found in all the films, whereas the content of W^5+^ increased sharply from 4.73% to 17.19%, in good agreement with the prediction by Niklasson et al.^[^ ^37^ ^]^ Furthermore, according to the ratio of W^6+^ and W^5+^, the chemical formulas can be derived as WO_2.97_, WO_2.95_, WO_2.93_, and WO_2.90_, suggesting that an increasing number of oxygen deficiencies were produced inside the as‐deposited films. This was verified by XPS O1*s* spectra which consisted of three characteristic peaks at 530.2 eV for W–O denoted by O_I_, 532.0 eV for oxygen vacancy denoted by O_II_, and 532.7 eV for absorbed oxygen denoted by O_III._^[^ ^43^ ^]^ As reflected by the O_II_ relative content increasing from 6.5% to 28.55%, a wide range of oxygen vacancy content was achieved. It should be noted that there have been some ambiguities in calibrating the absolute O/W content in the films and thus these ratios do not necessarily correspond to the actual stoichiometry of the specimens. However, it has been inferred that the oxygen vacancy content of the as‐deposited WO_3−*y*_ films varied over a wide range with the tuning of the Ar/O_2_ flow rate, which is in agreement with the recent findings by Shi and co‐workers.^[^ ^44^ ^]^ The Raman spectra (Figure 1c) showed that the generated W^5+^ ions formed W^5+^—O and W^5+^—W^5+^ bonds. With the increasing oxygen deficiency, the appearance of the as‐deposited WO_3−*y*_ films changed from transparent to completely black, and their optical transmittance significantly decreased from 73% to 3% at the measurement wavelength of 550 nm, as shown in Figure 1d.

**Figure 2:**
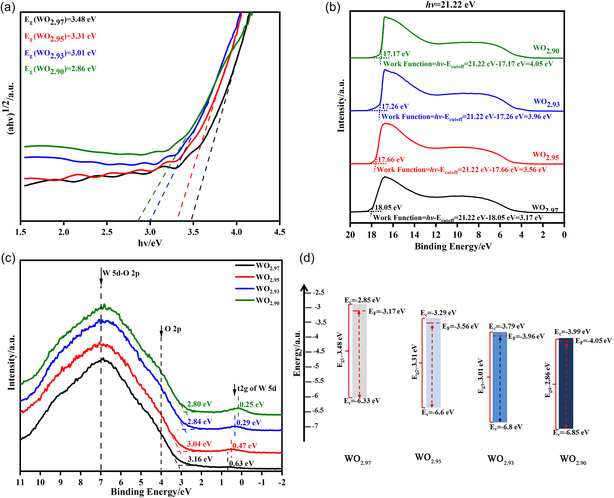
Band structure and electronic‐state analysis of tungsten oxide films with different oxygen vacancy concentrations. a) Optical bandgaps *E*_g_. b) Work functions by UPS tests. c) Valence band spectra obtained through XPS, d) Schematics of the energy band structure.

**Figure** **2** shows the electronic performances of the as‐deposited WO_3−*y*_ films. The optical bandgaps deduced from the transmittance spectra (Figure 1d) were 3.48, 3.31, 3.01, and 2.86 eV, respectively, for WO_2.97_, WO_2.95_, WO_2.93_, and WO_2.90_, showing a decreasing trend (Figure 2a). This suggests that more impurity energy levels are introduced with the increasing oxygen vacancy concentration. Combining the ultraviolet photoelectron spectroscopy (UPS) spectra (Figure 2b) and the XPS valence band spectra (Figure 2c),^[^ ^45^ ^]^ band structures of these as‐deposited WO_3−*y*_ films are depicted in Figure 2d, where the location of *E*_F_ was determined by the work function (shown in Figure 2b). The location of the valence band top, *E*_v_, was determined by *E*_v_–*E*_F_ (shown in Figure 2c). The location of the conduction band bottom, *E*_c_, was determined by *E*_g_ = *E*_c_ − *E*_V_ (shown in Figure 2a). Two observations were made: first, in the valence band spectra (Figure 2c), the t2*g* band of W5*d* shifted toward the Fermi level (denoted at 0 eV) from 0.63 to 0.25 eV, and the band peak became wider and stronger, suggesting that with the increasing oxygen vacancies, more electrons were introduced into the t2*g* band and became weakly bonded with the tungsten ions. Second, *E*_C_−*E*_F_ was calculated from Figure 2d (shown in Figure S4, Supporting Information). The free‐carrier concentration is given by the equation $E_{C}-E_{F}\text{ = }kT\text{ln}\left(\frac{N_{C}}{N_{D}}\right)$ for n‐type semiconductor WO_3_ (where *N*_C_ represents the conduction band effective density of states, *N*_D_ represents the carrier concentration, *k* is the Boltzmann constant, and *T* is the temperature). *E*_C_–*E*_F_ decreased with increasing oxygen vacancy content, demonstrating that more free electrons were introduced into WO_3−*y*_. In summary, the ionized oxygen vacancies act as double‐electron donors.^[^ ^46^ ^]^ Part of these donated electrons become free electrons in extended states, whereas others are localized, both of which are responsible for transforming the as‐deposited WO_3−*y*_ film from a transparent state to an absorbing state (as shown in Figure 1d).

This conclusion was further verified by the change in film resistance from 7.12 × 10^3^ Ω for W_2.90_ to 1.69 × 10^9^ Ω for WO_2.97_, an increase of six orders of magnitude, where the resistance was measured on films with the same geometric dimension (Figure S5, Supporting Information).

**Figure 3:**
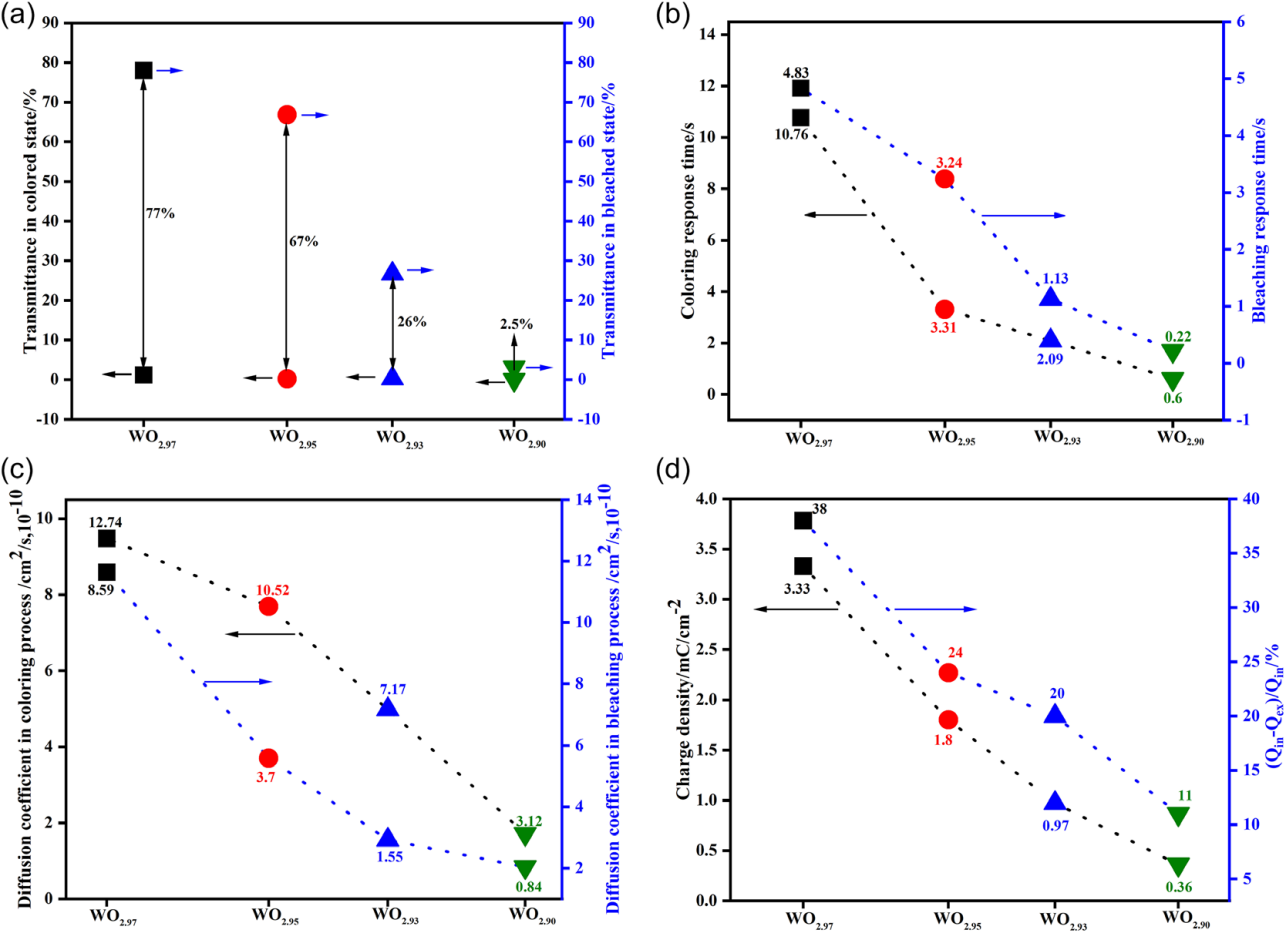
Initial EC properties of tungsten oxide films with different oxygen vacancy concentrations. a) Modulation rates at 550 nm. b**)** Response times. c) Diffusion coefficients of lithium ions. d**)** Charge densities *Q* and the “the trapping effect” of lithium ions illustrated by *Q*_ex_/*Q*_in_, where *Q*_ex_ indicates the extracted charge and *Q*_in_ represents the inserted charges during the EC process.

The initial EC and electrochemical properties were recorded and are shown in **Figure** **3**, with the original testing data provided in the Figure S6 and S7, Supporting Information. During the EC process, all the WO_3−*y*_ films became fully colored with the transmittance near 0% at 550 nm but not all of them could be bleached transparent, showing a decreasing transmittance modulation rate from 77% to 2.5% (Figure 3a and S6, Supporting Information). This demonstrates that oxygen vacancies in the WO_3−*y*_ films affect their color‐changing capability, which is the basis of selecting EC materials. Moreover, the color‐changing ability became weaker with the increasing number of oxygen vacancies. Additionally, the transmittance in the bleached state of all the WO_3−*y*_ films was higher than that in the as‐deposited state after the first bleaching (Figure S6, Supporting Information).

The response times determined from the current–time curves (Figure S7, Supporting Information), during both the coloring and bleaching processes, linearly decreased with increasing oxygen vacancies (Figure 3b). This improvement in the response time came at the cost of a sharp decrease in the transmittance modulation rate (Figure 3a), rendering the improvement unattractive. Notably, in comparison with the coloring and bleaching response times of WO_2.97_, that of WO_2.95_ were greatly improved from 10.76s/4.83s to 3.31s/3.24s, with a moderate decrease in the corresponding transmittance modulation rate from 77% to 67% (Figure 3a), which is beneficial for fast coloring response. Surprisingly, the diffusion coefficients of the inserted Li ions during both the coloring and bleaching processes (deduced from the CV curves at different scanning rates, as shown in Figure S8, Supporting Information) simultaneously showed sharp decreasing trends, which made it difficult to interpret the improvement in response time kinetics. However, with the increasing oxygen vacancy concentration, the storage charge density (calculated from CV curves shown in Figure S9, Supporting Information) greatly decreased from 3.33 to 0.36 mC cm^−2^. This was probably the main reason for the aforementioned improvement in response times of the WO_3−*y*_ films (Figure 3b). Interestingly, we calculated the *Q*_ex_/*Q*_in_ value from CV curves (shown in Figure S9, Supporting Information) that denotes the trapping effect and found it to be greatly increased from 62% to 89%, implying that fewer Li ions would be trapped during the EC process for a higher number of oxygen vacancies.

**Figure 4:**
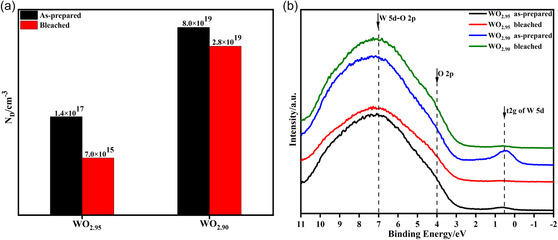
Explanation of the modulation rate variations of tungsten oxide films with different oxygen vacancy concentrations. a) Carrier concentrations and b**)** XPS valence band spectra of WO_2.95_ and WO_2.90_ in their as‐prepared and bleached states.

We explored the underlying mechanisms for the observed behaviors. **Figure** **4** shows the carrier concentrations (determined by capacitance–voltage and Mott–Schottky tests, as shown in Figure S10, Supporting Information) and the valence band spectra of WO_2.95_ and WO_2.90_ in their as‐deposited and bleached states. Here, the bleached state implies that the as‐prepared WO_3−*y*_ films were directly subjected to a positive bias of 3 V for 3 min without any coloring or Li‐ion insertion. Further, in the as‐prepared state, the carrier concentration of WO_2.90_ was more than two orders of magnitude higher than that of WO_2.95_, which was in good agreement with the results of the electronic analysis (Figure 2) and the electrical tests (Figure S5, Supporting Information). Compared to the as‐prepared samples, the carrier concentration decreased after the bleaching operation, which can be attributed to the disappearance of the electrons located in the t2*g* bands of WO_2.95_ and WO_2.90_, as shown in the XPS valance bands (Figure 4b). This was also verified from the analysis of W4*f* and O1*s* XPS spectra shown in Figure S11, Supporting Information, where obvious decreases in the concentration of both W^5+^ and O_II_ were observed in comparison with that in the as‐prepared samples (Figure 1a,b, 8.98% W^5+^ and 14.5% O_II_ for WO_2.95_; 17.19% W^5+^ and 28.55% O_II_ for WO_2.90_) with the bleached ones (Figure S11, Supporting Information, 7.82% W^5+^ and 8.61% O_II_ for WO_2.95_; 7.20% for W^5+^ and 11.10% for O_II_ for WO_2.90_). The disappearance of the t2*g* band electrons was also responsible for the increased transmittance in all the WO_3−*y*_ films after bleaching during the first EC process (Figure S6, Supporting Information).

Furthermore, we note that a greater decrease of the carrier concentration was found in WO_2.90_, from 8.0 × 10^19^ to 2.8 × 10^19^ cm^−3^ in comparison with that in WO_2.90_ from 1.4 × 10^17^ to 7.0 × 10^15^ cm^−3^, because there were much more electrons existing in the t2*g* band for WO_2.90_ than in WO_2.95_, in the as‐prepared state (Figure 4b). After the bleaching operation, the left carriers became the main free electrons and because of the intensive reflection by these free carriers (2.8 × 10^19^ cm^−3^), the transmittance of the bleached WO_2.90_ remained low at 2.5% at 550 nm (Figure S6, Supporting Information), and only 2.5% optical modulation rate was achieved (Figure 3a). While for WO_2.95_, the transmittance in the bleached state reached 70% and the optical modulation rate reached 67% originating from its low free‐electron concentration (7.0 × 10^15^ cm^−3^). These results first experimentally demonstrated that only the intrinsically located or externally introduced electrons in the t2*g* band of W5*d* contribute to the formation of color centers and could be manipulated by an electric field. These electrons render the EC characteristics and conductivity to tungsten oxide. However, the free electrons introduced by oxygen vacancies degrade the coloring‐changing capability. These results are in good agreement with Faughnan's theoretical prediction.^[^ ^47^, ^48^ ^]^

**Figure 5:**
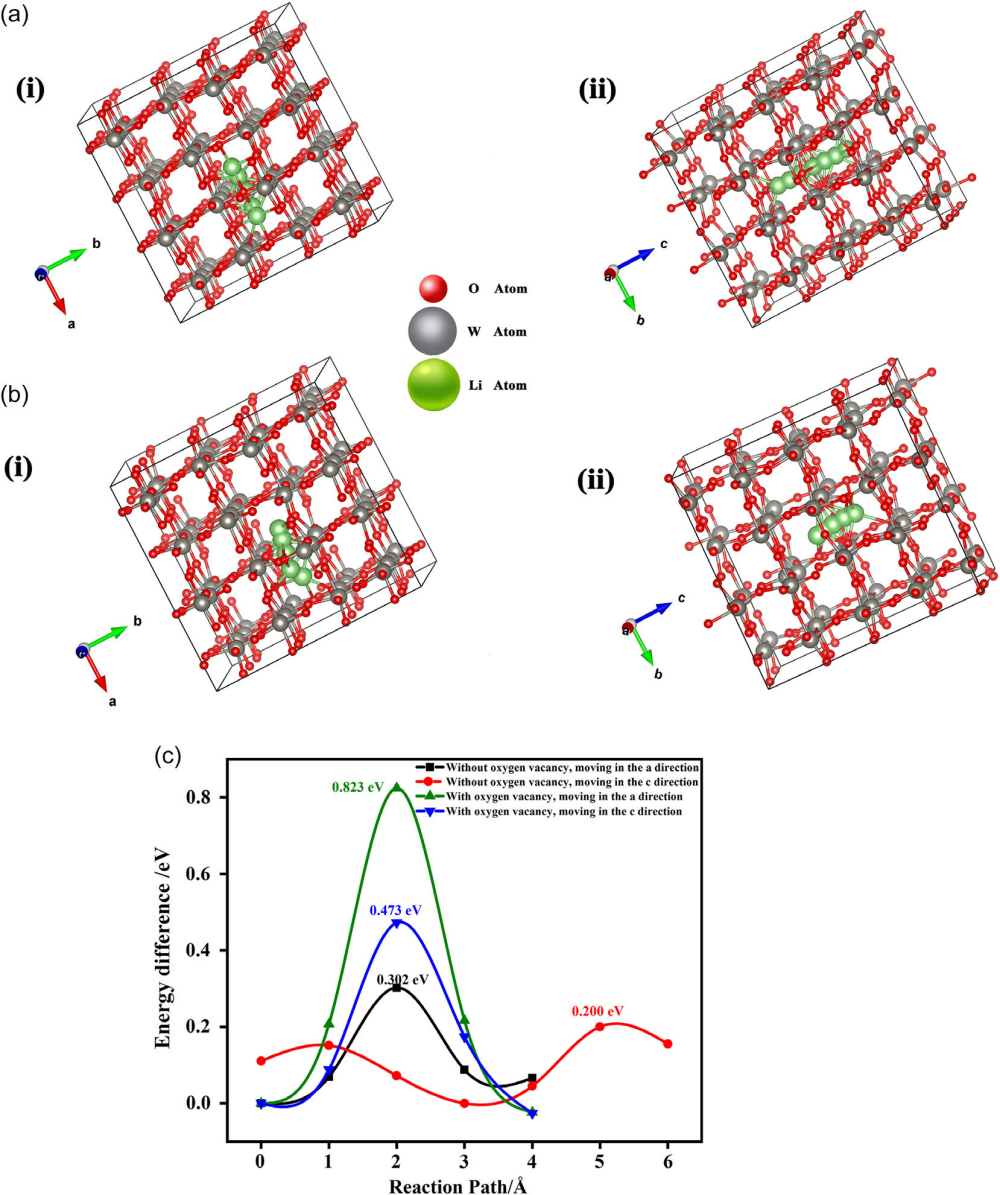
First‐principle calculations on the diffusion of lithium ions. a) Diffusion paths of lithium ions in the absence of oxygen vacancies. b) Diffusion paths of lithium ions in the presence of oxygen vacancies. c) Calculated diffusion barriers for lithium ions along different directions and in various states.

The transport kinetics of the inserted Li ions in the monoclinic WO_3−*y*_ host with and without oxygen vacancies were analyzed using DFT calculations, as shown in **Figure** **5**.

Owing to the symmetric structure along the a and b directions, diffusion paths along a and c were chosen, as illustrated in Figure 5a,b. Calculation results demonstrated that upon introduction of oxygen vacancies in the supercell, the diffusion barriers of the inserted Li ions increased significantly to 0.823 from 0.302 eV in the *a*‐direction and to 0.473 from 0.200 eV in the *c*‐direction. This is probably because the introduced oxygen vacancies distort the WO_6_ octahedral cage, drive the W ion away from its high‐symmetry position, and distort the octahedral channels of Li‐ion transport.^[^ ^33^ ^]^ These higher diffusion barriers were responsible for the continuously decreasing diffusion coefficients during both the coloring and bleaching processes with increasing concentrations of oxygen vacancies (Figure 3c).

**Figure 6:**
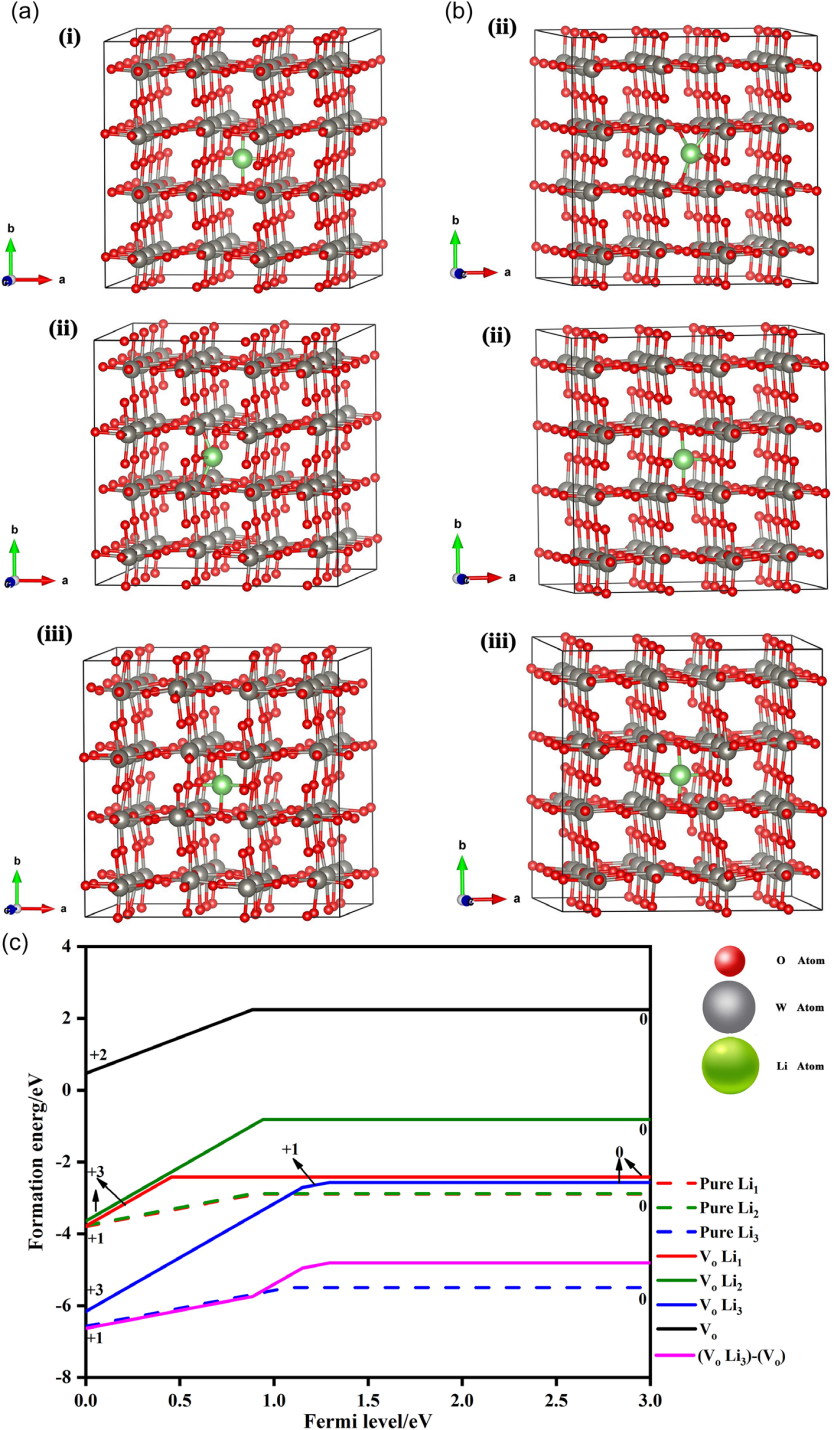
First‐principle calculations on the formation energy of lithium ions. a) Model diagram illustrating insertion of lithium ions into tungsten oxide lattice without oxygen vacancies. b) Model diagram illustrating insertion of lithium ions into tungsten oxide lattice in the presence of oxygen vacancies. c) Line charts of the formation energy of lithium ions in tungsten oxide lattices in different defect states.

The formation energies as a function of the Fermi level *E*_F_ are shown in Figure **6**. The slope of each curve corresponds to the charge state of the defects. Considering the two types of defects, including inserted Li ions and oxygen vacancies, and their mutual superposition and possible existing states that are neutral (Li^0^), singly charged (Li^+^), and neutral (V^0^), singly charged (V^+^), or doubly charged (V^2+^) for oxygen vacancies, six possible models were established, and their corresponding formation energies were calculated (Figure 6a,b). The calculation results are shown in Figure 6c. In the undisturbed lattice, only singly charged Li^+^ exists and the formation energy of pure Li_3_ is the lowest at −5.5 eV. Once an oxygen vacancy is introduced into the lattice, it is unique in the +2 charge state, which agrees with Wang's calculation.^[^ ^49^ ^]^ The formation energy of pure doubly charged (V^2+^) is the highest above 0 eV, which suggests that oxygen vacancies cannot remain unperturbed when Li ions are inserted. In the superposition between the oxygen vacancies and inserted Li ions, the formation energy of VoLi_3_ is the lowest, at −6.2 eV, which is 0.7 eV higher than pure Li_3_ when oxygen vacancies are absent in the lattice. These results suggest that the introduced oxygen vacancies are unique to the +2 charge state and are electrostatically repulsive to the inserted Li^+^ ions. The large Coulomb repulsion makes the state of the inserted Li ions less stable (denoted by the higher formation energy) and easier to extract, but harder to inject. This is why the storage charge density decreases, whereas the trapping effect is attenuated with increasing oxygen vacancy content (Figure 3d). A mechanistic understanding of the trapping effect passivation of oxygen vacancies provides key insights into the enhancement of the cyclic stability for most transition metal oxides.

**Figure 7:**
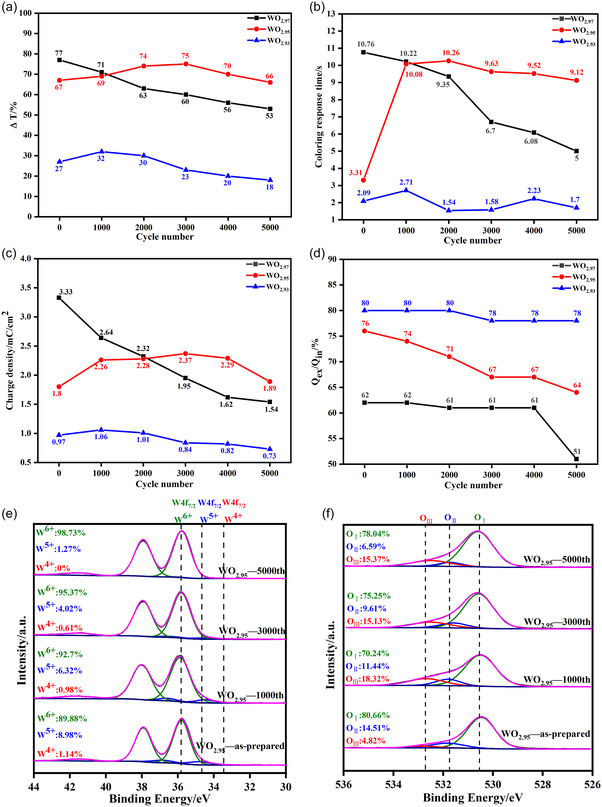
Cyclic characters of tungsten oxide films with different oxygen vacancy concentrations. a) Charge density *Q*. b) Trapping effect of lithium ions denoted by *Q*_ex_/*Q*_in_. c**)** Modulation rate at 550 nm. d) Coloring response time, and XPS analysis of W and O elements in WO_2.95_ film during cycling. e) W element. f) O element.

A total of 5000 CV cycles were performed on WO_2.97_, WO_2.95_, and WO_2.93_ films, and the WO_2.90_ sample was discarded because of its rather weak color‐changing capability for tracing. The cyclic stabilities of the electrochemical and EC properties of the three WO_3−*y*_ films were recorded using the original test data shown in Figure S11–S17, Supporting Information, and the analyzed data are summarized in **Figure** **7**a–d. It can be seen that, for WO_2.97_, the charge density monotonically decreased from 3.33 to 1.54 mC cm^−2^; for WO_2.95_, it first increased from 1.8 mC cm^−2^ to the maximum of 2.37 mC cm^−2^ after 3000 CV cycles and then decreased to 1.89 mC cm^−2^ after 5000 CV cycles with 100% capacity retention; for WO_2.90_, the charge‐density top value was obtained at 1.06 mC cm^−2^ after 1000 CV cycles and then decreased 0.73 mC cm^−2^ after 5000 CV cycles (Figure 7a). As to “the trapping effect” denoted by *Q*_ex_/*Q*_in_ over the 5000 CV cycles, a complex situation was observed (Figure 7b). For WO_2.97_, the factor remained stable around 61–62% in the first 4000 CV cycles and then decreased sharply to 51% after the last 1000 CV cycles, suggesting that a sudden aggravation of the trapping effect occurred to the inserted Li ions, which also implies acceleration of the electrochemical failure of the tungsten oxide film. For WO_2.95_, *Q*_ex_/*Q*_in_ first slowly decreased from 76% to 67% in the first 3000 CV cycles and then remained stable during the next 1000 CV cycles and then again gradually decreased to 64% after 5000 CV cycles. For WO_2.93_, *Q*_ex_/*Q*_in_ remained stable and high around 78–80%. It should be noted that during long‐term cycling, *Q*_ex_/*Q*_in_ for WO_2.95_ remained higher than that for WO_2.97_, indicating that the passivation role of oxygen vacancies in “the trapping effect” is significant.

Interestingly, during 5000 CV cycles, the transmittance modulation rate (Figure 7c) showed approximately the same variation trend as the charge density (Figure 7a). For WO_2.97_, the modulation rate at 550 nm monotonically decreased from 77% to 53% after 5000 CV cycles, whereas for WO_2.95_ and WO_2.93_, the modulation rates first increased and then decreased. The value became the highest (75%) after 3000 CV cycles for WO_2.95_ and (32%) after 1000 CV cycles for WO_2.90_. Notably, the transmittance modulation rate of WO_2.95_ after 5000 CV cycles was approximately the same as that in the first cycle, suggesting that 100% retention was achieved (Figure 7c). Compared with the relatively fast and stable response times during bleaching processes (summarized in Figure S17, Supporting Information), the coloring response time monotonously decreased from 10.76 to 5 s for WO_2.97_; for WO_2.93_, the change was small ranging from 1.54 s at the 2000th cycle to 2.71 s at the 1000th cycle; while for WO_2.95_, the coloring response time increased sharply during the first 1000 CV cycles from 3.31 to 10.08 s and then exhibited a gradually decrease from 10.26 to 9.12 s (Figure 7b). The synchronous variation trends between the coloring response time and charge density over the 5000 CV cycles proved that the improvement in coloring and bleaching response times with increasing oxygen vacancies originated from the decreasing charge density (Figure 3b). These results demonstrate that the storage charge density is of decisive significance for both the optical modulation and response characteristics of the tungsten oxide films. Notably, when both WO_2.97_ (2.32 mC cm^−2^ around the 2000th CV cycle) and WO_2.95_ (2.37 mC cm^−2^ around the 3000th CV cycle) exhibited approximately the same charge density, WO_2.95_ showed a much higher transmittance modulation rate (75% vs 63%) and the similar coloring response time (9.63 vs 9.35 s). This shows that WO_2.95_ yielded a higher coloration efficiency and equivalent response times in comparison with WO_2.97_ film.

All these variations were related to the evolution of the oxygen vacancy content during the 5000 CV cycles. Figure 7e–f representatively shows the evolution of oxygen vacancy content in WO_2.95_. It can be seen that contents of O_II_ associated with the oxygen vacancy kept decreasing from 14.51% to 6.59% over the 5000 CV cycles. Notably, the value after 5000 cycles is equivalent to that in the as‐deposited WO_2.97_ film (at 6.51%), as shown in Figure 1b. This is why all the EC and electrochemical properties of WO_2.95_ improved or stabilized during the first 4000 CV cycles and then slowly degraded in the last 1000 CV cycles, comparable to the behavior of WO_2.97_ in long‐term cycling (Figure 7a–d). These results demonstrate the crucial role of oxygen vacancies in determining the cyclic stability of transition metal oxide films in terms of their electrochemical behaviors.

## 3 Conclusions

This study presented a comprehensive understanding of the multiple roles of oxygen vacancies in regulating the intrinsic optical, electrical, electronic, and bonding characteristics, EC properties, transport kinetics of inserted ions, and cyclic stability of amorphous WO_3−*y*_ films. Furthermore, the feasibility of passivating “the trapping effect” via tuning oxygen vacancies was investigated. The oxygen vacancy content was widely varied from 6.5% to 28.5%. With the increasing oxygen vacancy concentration, the following observations were made. 1) More W^5+^ ions were produced and a massive number of electrons were introduced, partly as free carriers and others entering the t2*g* band of W5*d* as weakly bonding electrons, both of which resulted in the improved conductivity of the as‐deposited WO_3−*y*_ films. 2) Due to optical diffraction and absorption of these two types of electrons, transmittance of the as‐deposited WO_3−*y*_ films at 550 nm sharply decreased from 73% to 3%, from transparent to completely black in appearance. 3) During the EC process, the optical modulation rate at 550 nm decreased from 77% to 2.5%, because only the intrinsically located or externally introduced electrons in the t2*g* band of W5*d* formed color centers and could be manipulated by an electric field. These electrons rendered the EC capability to tungsten oxide and the introduced free electrons greatly eroded the bleached‐state film transmittance. 4) Regarding electrochemical properties, the stored charge density and diffusion coefficients of the inserted ions during both coloring and bleaching processes were greatly attenuated, due to the larger diffusion barrier, higher formation energy of the inserted ions in the lattice, and electrostatic repulsion from the oxygen vacancies in the +2 charge state. 5) Because of the lower stored charge density, higher formation energy, and electrostatic repulsion from the oxygen vacancies in the +2 charge state, the trapping effect of the inserted ions was greatly attenuated, as reflected by the increase in *Q*_ex_/*Q*_in_ rate from 62% to 89%. 6) During the 5000 CV cycles, oxygen vacancy concentration decreased and perfect cyclic stability was achieved in WO_2.95_ with 100% retention of the optical modulation rate and stored charge density.

In summary, the presence of the oxygen vacancy in amorphous WO_3−*y*_ films is detrimental to their EC properties, especially the color‐changing capability, but it significantly attenuates “the trapping effect” and improves their cyclic stability. This suggests a trade‐off between these two aspects with an optimal oxygen vacancy content, which was found to be ≈14.5% for amorphous WO_3−*y*_ films. Our pioneering findings provide an effective solution to passivate “the trapping effect” of inserted ions that ubiquitously exist in transition metal oxide electrodes and greatly improve their cyclic stability. We also resolved the issue concerning the controversial roles of oxygen vacancies in determining EC behaviors, which is of great importance for defect engineering and science.

## 4 Experimental Section

4.1

4.1.1

##### Preparation of WO_3−y_ Films

Indium tin oxide (ITO)‐coated glass substrates (overall thickness ≈1.1 mm, sheet resistance ≈6 Ω sq.^−1^) were purchased from Zhuhai Kaivo Optoelectronic Technology Co. Ltd. China and cleaned successively with acetone, alcohol, and deionized water before use. WO_3−*y*_ films were fabricated by DC reactive magnetron sputtering using a JCP‐500 sputtering system (Technol.cn) and a metal tungsten target with a purity of 99.999%, having dimensions of 3 mm in thickness and 3 inches in diameter. The target–substrate separation was 110 mm. The background pressure was 6.0 × 10^−4^ Pa, the working pressure was 0.9 Pa, and the sputtering power was 80 W. The Ar/O_2_ ratio was adopted to regulate the oxygen vacancy content in as‐prepared WO_3−*y*_ films, which was set as 16 sccm:6 sccm, 25 sccm:3 sccm, 35 sccm:3 sccm, and 40 sccm:3 sccm. The substrate temperature was kept at ≈25 °C. To maintain a constant film thickness, the deposition time was tuned from 0.75 to 1.5 h. Meanwhile, the 1 mol L^−1^ Li‐ion electrolyte was prepared by dissolving LiClO_4_ in a polycarbonate (PC) solution.

##### Characterization and Performance Tests

The film thickness was tested using a step profiler (Dektak XT Bruker), and all the prepared WO_3−*y*_ films were ≈550 nm thick (as indicated by the cross‐sectional scanning electron microscopy (SEM) images in Figure S1, Supporting Information). Crystallization of the WO_3−*y*_ films was tested by X‐ray diffraction (XRD) analysis using Cu K*α* radiation (Philips X’Pert diffractometer). The surface morphology was studied via SEM using a Sigma 500 instrument (Zeiss). The bonding characteristics were determined using Raman spectroscopy (LABRAM HR EVOLUTION, HORIBA). X‐ray photoelectron spectroscopy (XPS) data and valence band spectra were recorded using an X‐ray photoelectron spectrometer (Thermo Fisher Scientific ESCALAB 250 XPS system). The work function was measured using a UV photoelectron spectrometer (Thermo Fisher Scientific ESCALAB 250 UPS System). Transmittance spectra were recorded using a UV‐vis spectrophotometer (Shimadzu UV 3150, Japan), and the optical bandgap *E*_g_ was calculated. During the optical properties test, all the samples were colored and bleached under ±3 V and the operation time was set to be 5 min to ensure that all samples were fully colored and bleached.

All electrochemical measurements were performed in a 1 m/LiClO_4_/propylene carbonate solution on an electrochemical workstation (CHI760E) using the WO_3−*y*_ films as the working electrode, a platinum sheet as the counter electrode, and Ag/AgCl as the reference electrode. Cyclic voltammetry (CV) and chronoamperometry (CA) measurements were carried out in the potential range from −0.8 to 0.8 V, and diffusion coefficients of inserted lithium ions in host WO_3−*y*_ were analyzed from CV characteristics at different scanning rates.^[^ ^50^ ^]^ The effective ion diffusion coefficients *D* for Li^+^ can be estimated from the Randles–Servcik equation as follows.

(1) $$\;D^{1/2}=\frac{i_{\text{Pa}/\text{Pc}}}{2\text{.69}\;\times \;10^{5}\;\times \;A\;\times \;C_{0}\;\times \;n^{3/2}\nu^{1/2}}$$

where *A* is the film area, *C*_0_ is the electrolyte concentration, and *n* is the number of electrons transferred. By analyzing the linear diagrams between the oxidation peak current (*i*_pa_) and reduction peak current (*i*_pc_) at different scanning rates and *v*1/2, the diffusion rate of lithium ions in the tungsten oxide films can be quantitatively determined. In addition, the charge capacities (*Q*_ex_ and *Q*_in_) were calculated by integrating the insertion and extraction parts of the CV data.^[^ ^28^ ^]^ The response times were determined from the CA curves. The electrical properties were investigated through Mott–Schottky analyses using an electrochemical workstation (CHI760E) in a 1 m/LiClO_4_/polycarbonate (PC) solution at an AC amplitude of 5 mV and a frequency of 1000 Hz. The film capacitance was obtained from a self‐assembled CV/IV testing platform (M‐4L, IVTEST.com) using a Keithley 2400 source meter.

##### DFT Calculations

Density functional theory (DFT) calculations were performed to simulate the insertion/extraction and diffusion behaviors of lithium ions in WO_3−*y*_ films with/without the existence of oxygen vacancies. All calculations were performed using the Vienna ab initio simulation package. The generalized‐gradient approximation (GGA)‐Perdew–Burke–Ernzerhof (PBE) functional was selected for the exchange and correlation potentials. Weak van der Waals interactions were considered by using the DFT‐D3 functional. The cut‐off energy for the plane wave was 500 eV for the diffusion process and 400 eV for the binding energy during the insertion/extraction process. The Gamma point in the Brillouin zone was chosen for integration. The total energies of the systems converged to 10^−5^ eV in the iteration solution of the Kohn–Sham equation. The force on each atom was reduced to 0.05 eV Å^−1^ after geometry optimization. The unit cell was set as 2 × 2 × 4 in a tetragonal system with a lattice constant of 15 Å.

## Conflict of Interest

The authors declare no conflict of interest.

## Supporting information

Supplementary Material

## Data Availability

The data that support the findings of this study are available in the supplementary material of this article.
