# Supplementary material for: The Counterbalancing Role of Oxygen Vacancy between the Electrochromic Properties and the Trapping Effect Passivation for Amorphous Tungsten Oxide Films
Source: Small Sci. 2024 Jan 20;4(3):2300219. doi: 10.1002/smsc.202300219 (PMC11935010; doi:10.1002/smsc.202300219)
Supplement: Supplementary file 1 — Supplementary Material [file SMSC-4-2300219-s001.pdf]

Supplementary information

The counterbalancing role of oxygen vacancy between the electrochromic properties and the trapping effect passivation for amorphous tungsten oxide films

Zhaocheng Zhang[1] , Huajing Mo[1], Ruicong Li [1], Xinglong Zhou[1], Zicong Lin [1], Jiong Zhang [2,*], Xiufeng Tang [1, 3, *], Yunfeng Zhan [1, 3 , *]and Jianyi Luo [1, 3, *]

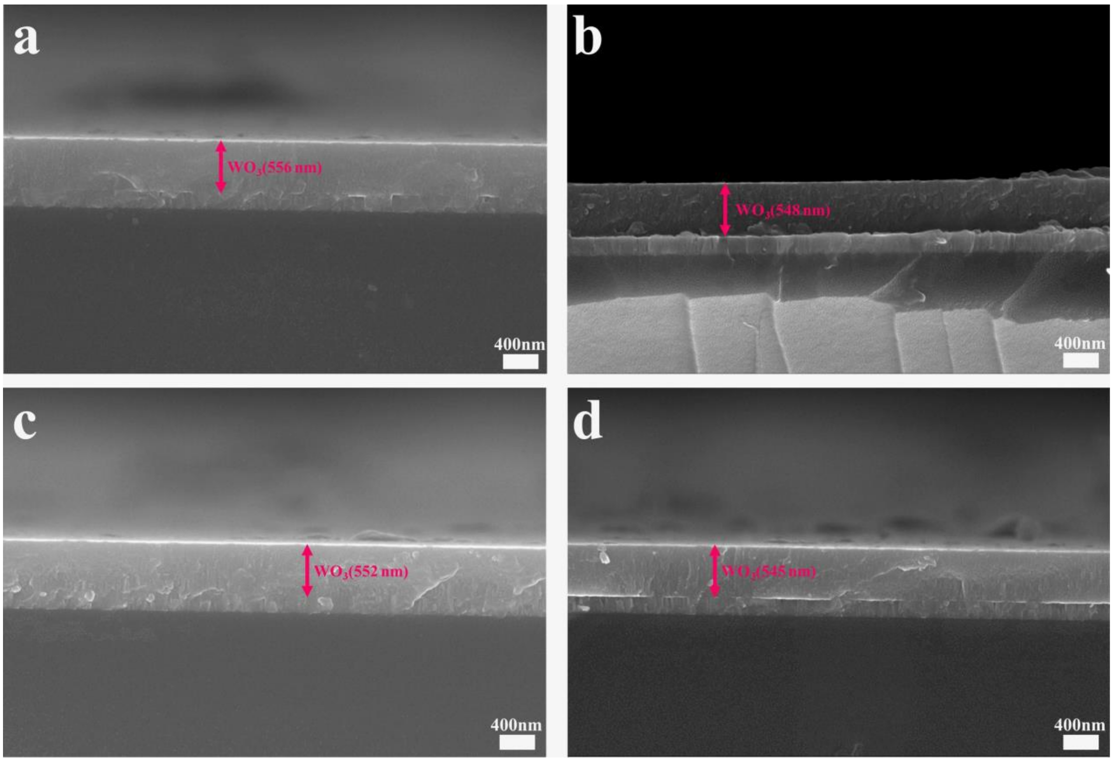

Fig.S1 Corss-sectional SEM images of the tungsten oxide films with different oxygen vacancies. **a.**$WO_{2.97}$, **b.** $WO_{2.95}$, **c.**$WO_{2.93}$, **d.**$WO_{2.90}$

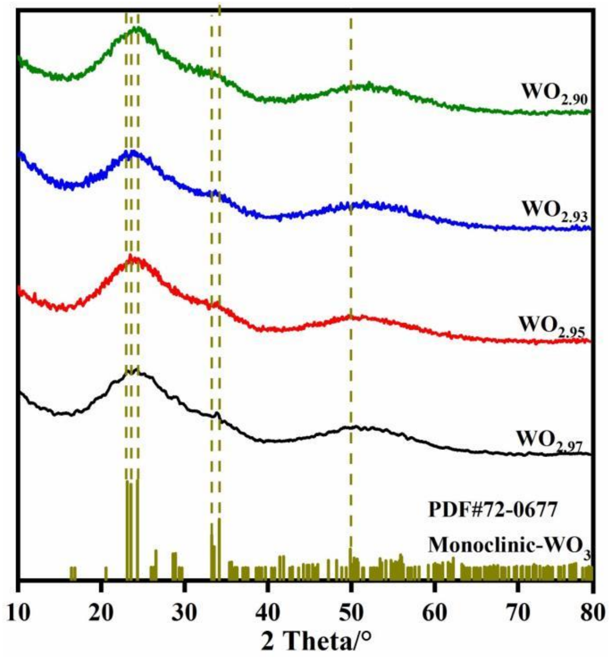

Fig.S2 XRD spectra of the tungsten oxide films with different oxygen vacancies

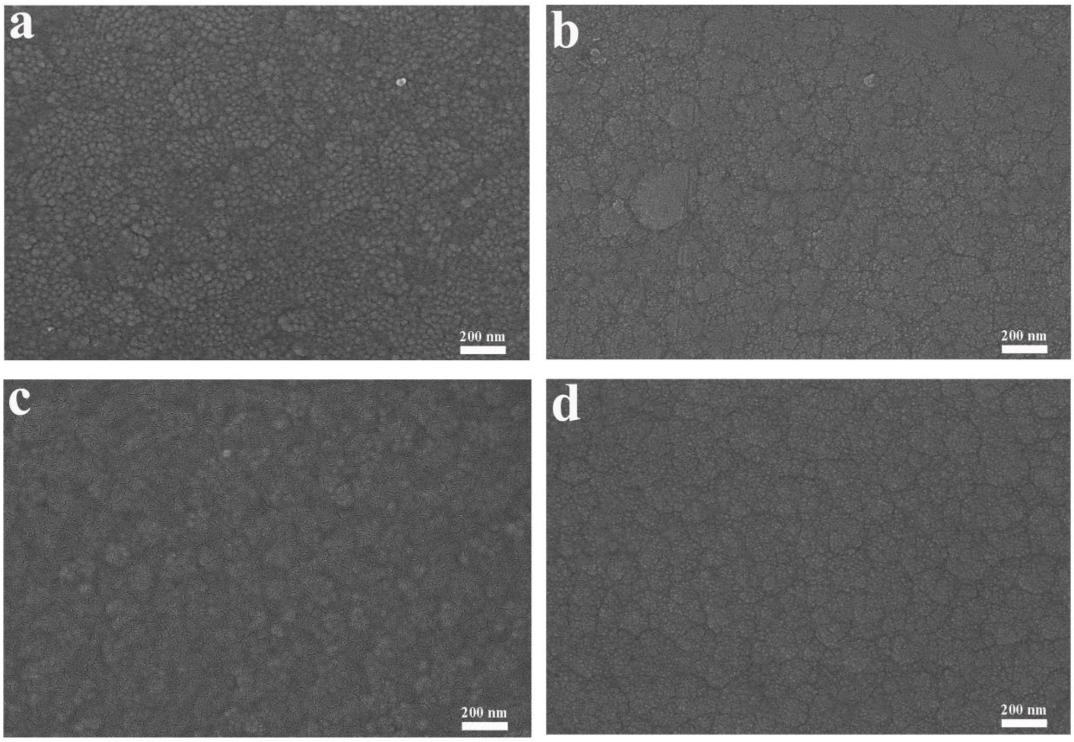

Fig.S3 SEM images of the tungsten oxide films with different oxygen vacancies.
a.$WO_{2.97}$, **b.** $WO_{2.95}$, **c.**$WO_{2.93}$, **d.**$WO_{2.90}$

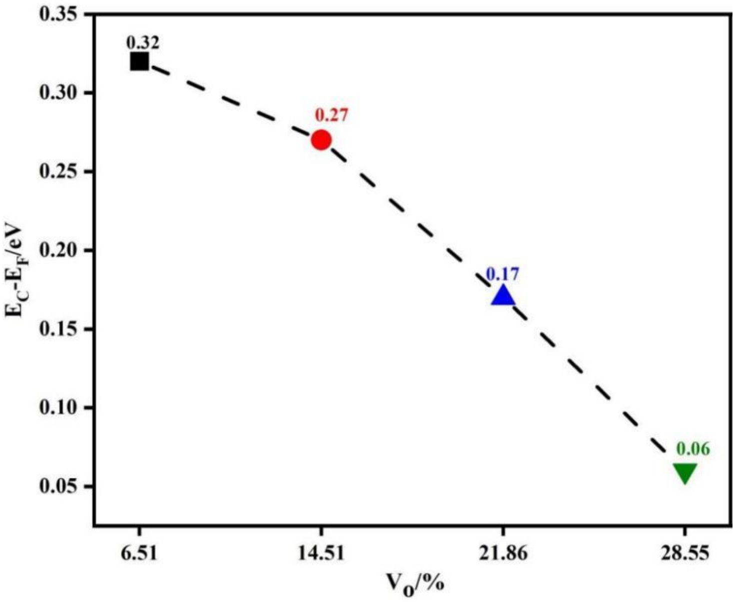

Fig.S4 E_C-E_F of tungsten oxide films with different oxygen vacancies

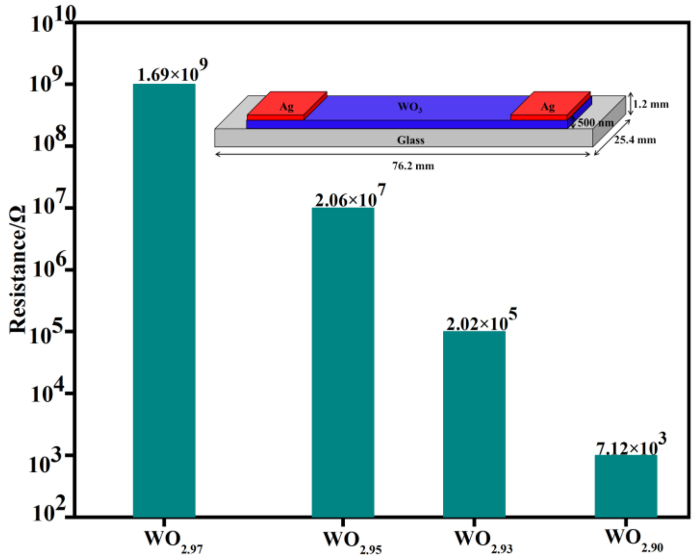

Fig.S5 Film resistances tested in the same geometric dimension shown by the inset illustration

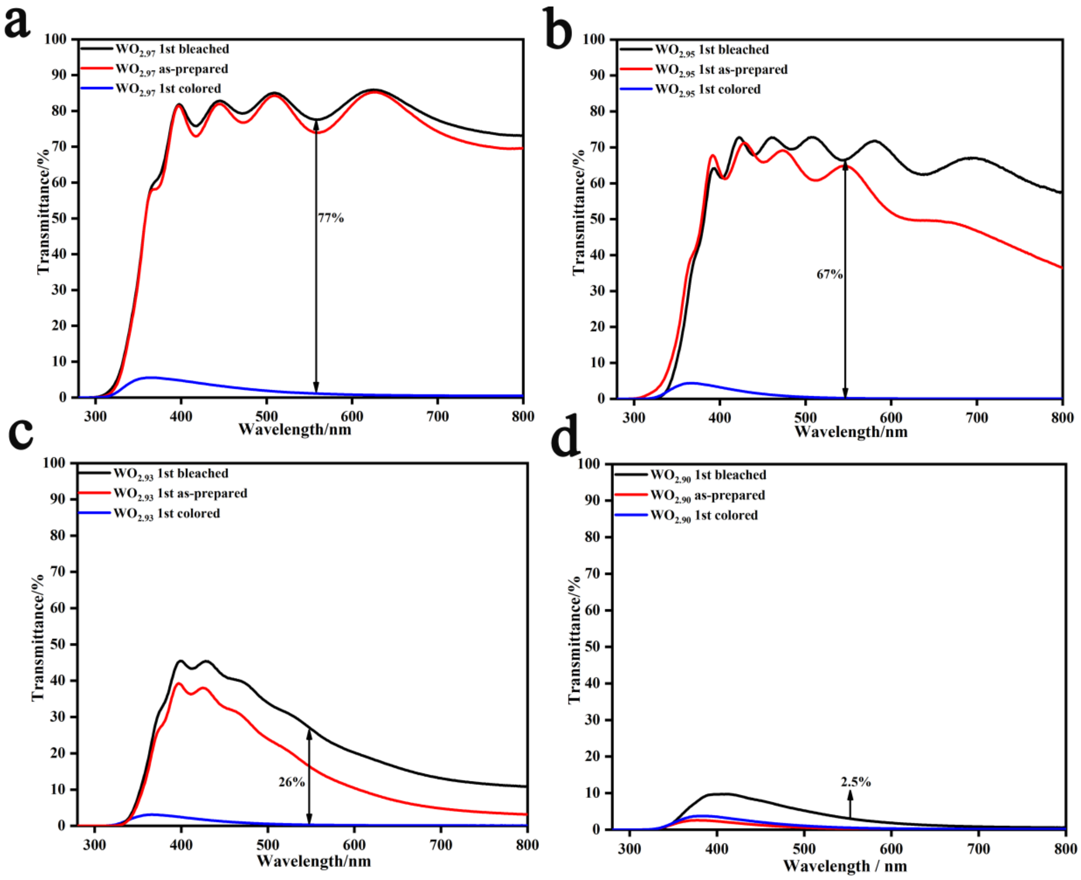

Fig.S6 Transmittance spectra of tungsten oxide films with different oxygen vacancies. **a.** $WO_{2.97}$, **b.** $WO_{2.95}$, **c.**$WO_{2.93}$, **d.**$WO_{2.90}$

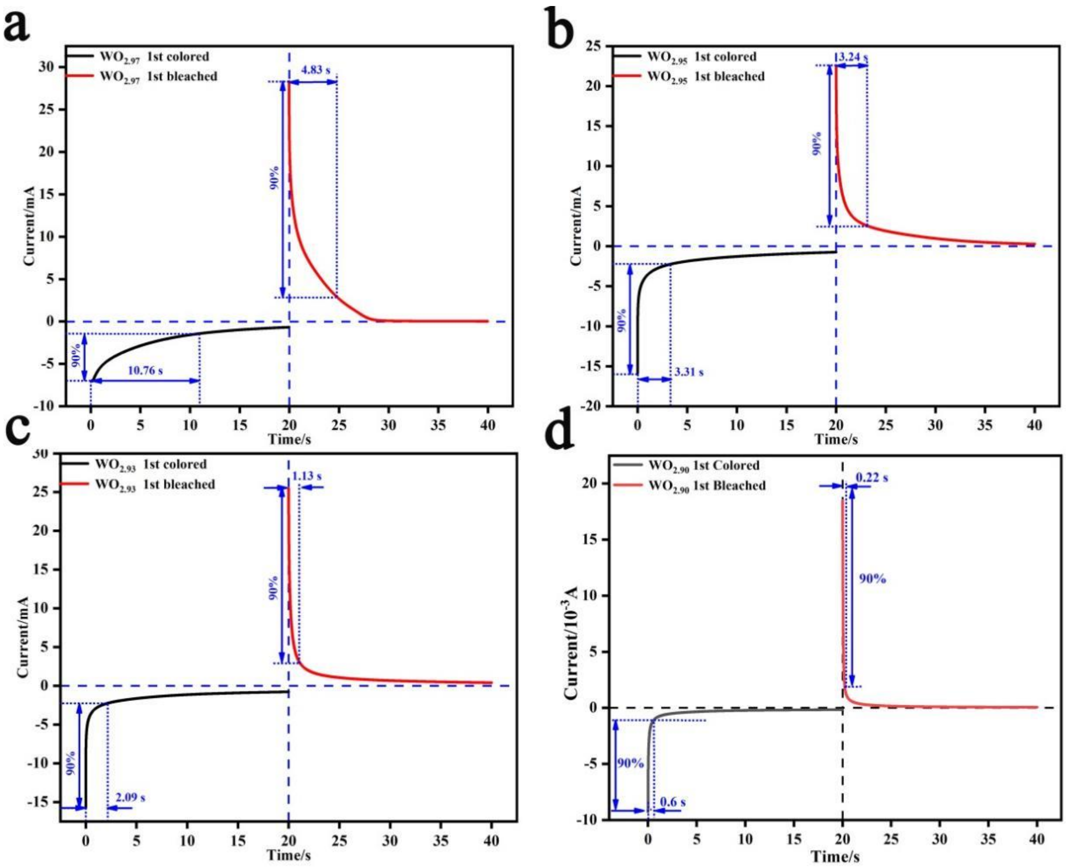

Fig.S7 Current-time curves to demonstrate the response time of tungsten oxide films with different oxygen vacancies. **a.** $WO_{2.97}$, **b.** $WO_{2.95}$, **c.** $WO_{2.93}$, **d.** $WO_{2.90}$

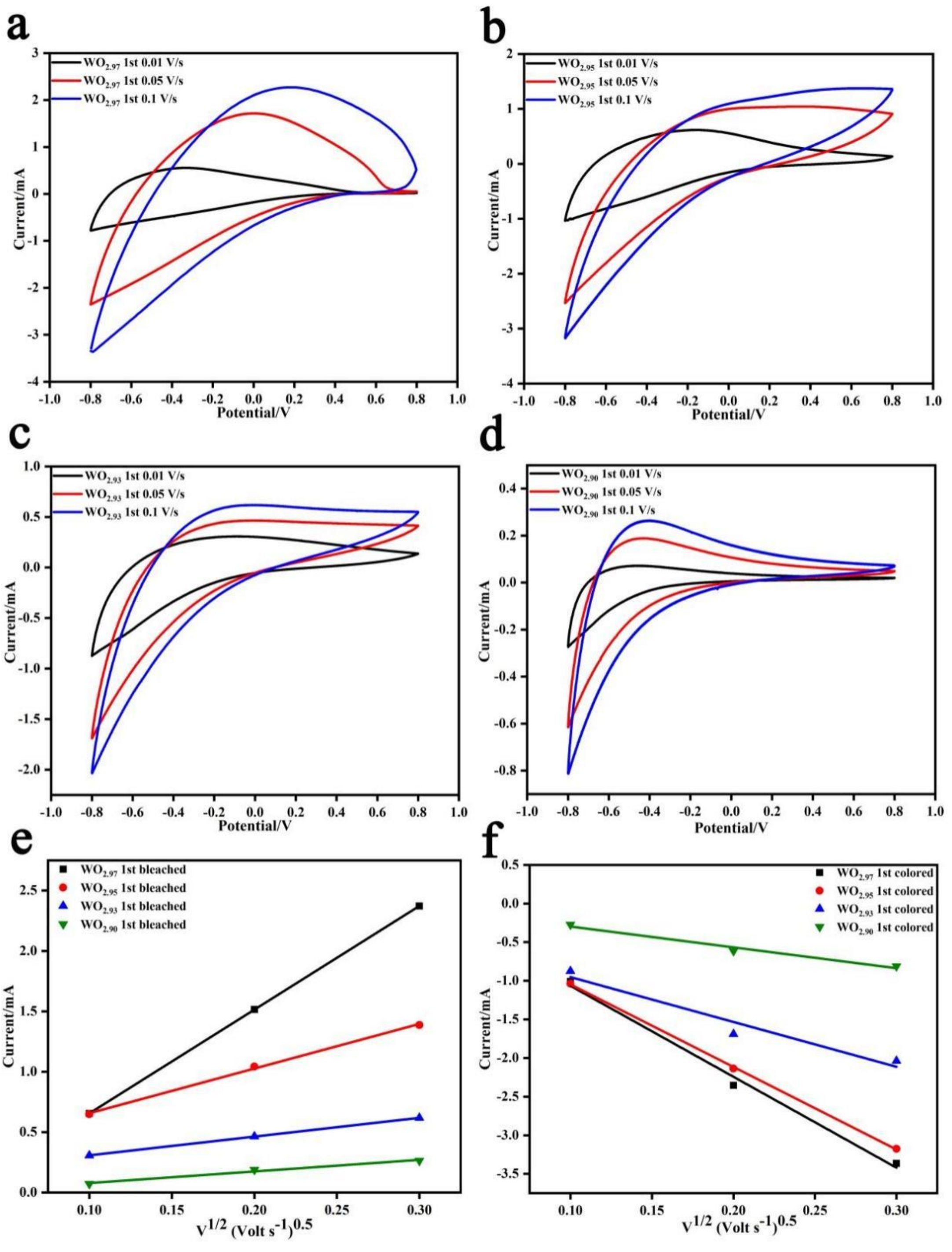

Fig.S8 CV curves at different scanning rates to calculate the diffusion coefficient of tungsten oxide films with different oxygen vacancies. **a.** $WO_{2.97}$, **b.** $WO_{2.95}$, **c.** $WO_{2.93}$, **d.** $WO_{2.90}$, **e**, **f.** the relationship between the oxidation/reduction peak current and the scanning rate $v^{1/2}$, respectively

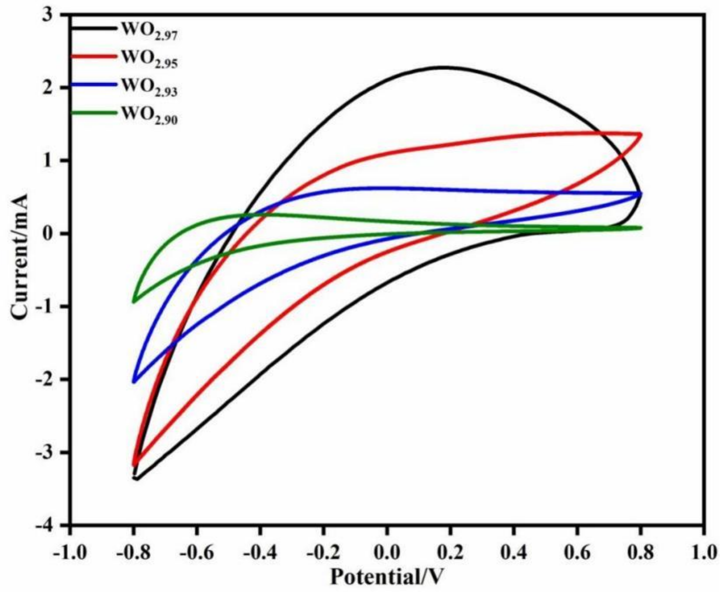

Fig.S9 CV curves of initial tungsten oxide films with different oxygen vacancies

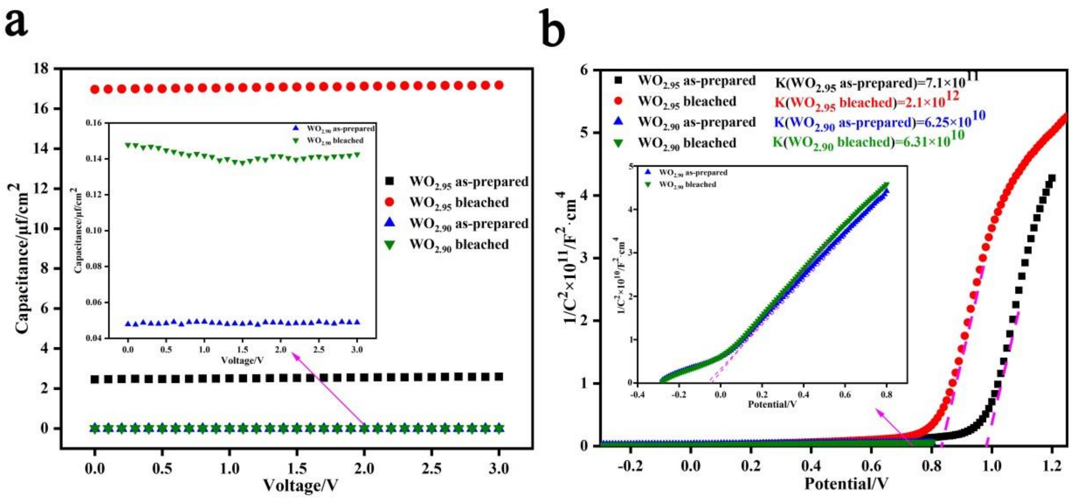

Fig.S10 Capacitance-voltage (**a**) and Mott-Schottky tests (**b**) to determine the carrier concentrations of tungsten oxide films with different oxygen-vacancy concentrations

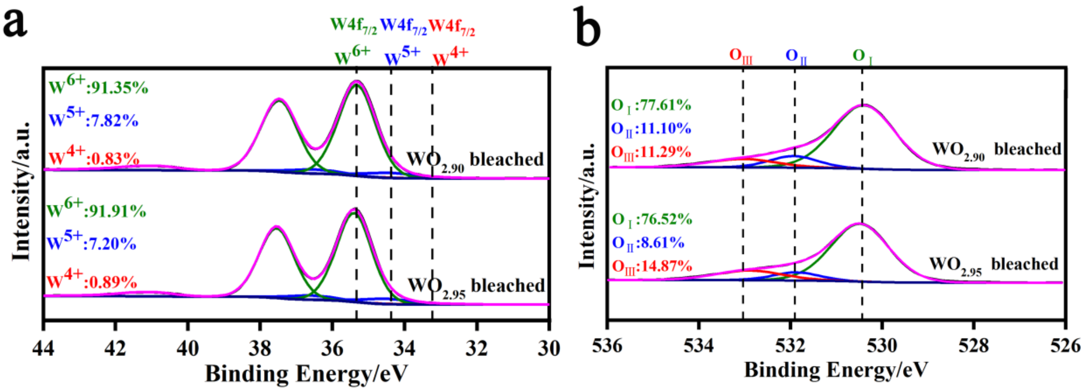

Fig.S11 XPS spectra of as-deposited $WO_{2.95}$ and $WO_{2.90}$ films after being bleached under a positive bias of 3 V lasting for 3 min. **a.** W element, **b.** O element

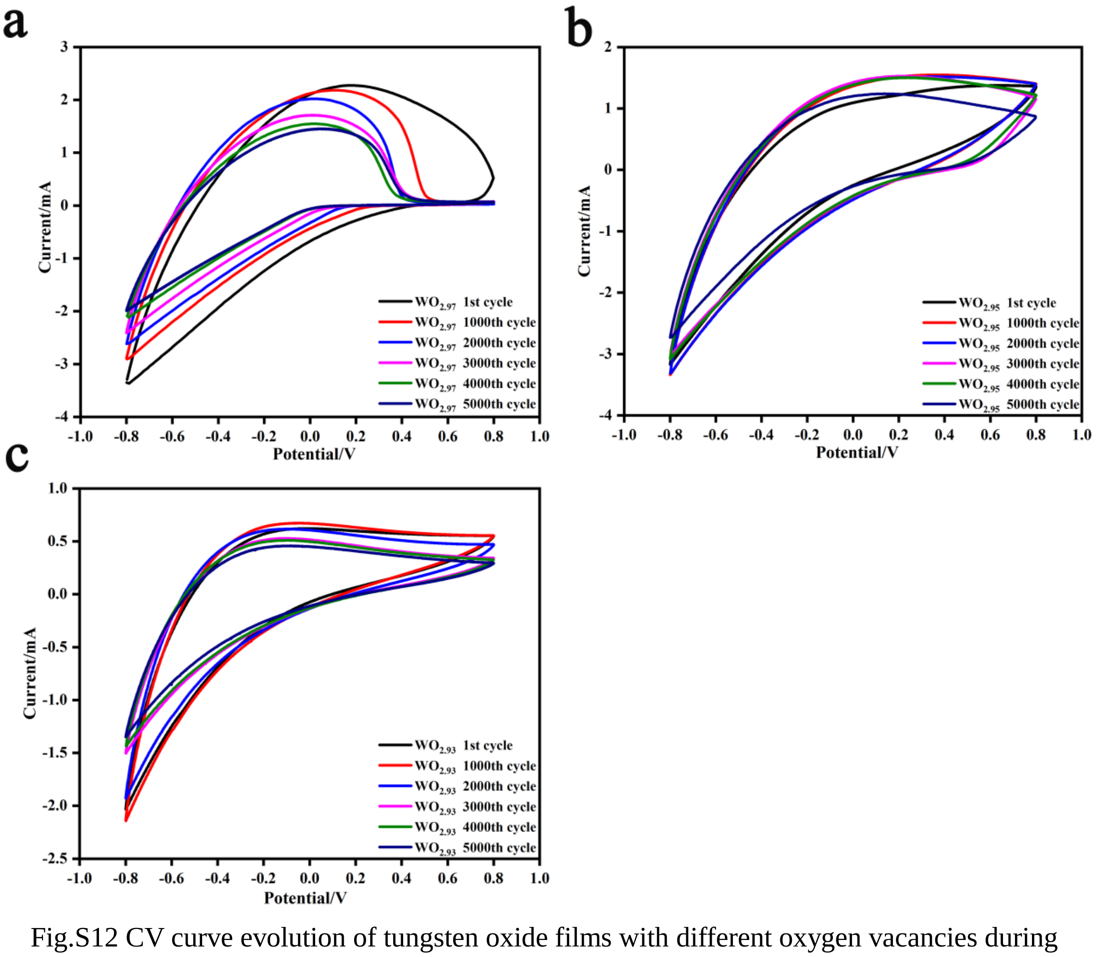

Fig.S12 CV curve evolution of tungsten oxide films with different oxygen vacancies during long-term cycling. **a.** $WO_{2.97}$, **b.** $WO_{2.95}$, **c.** $WO_{2.93}$

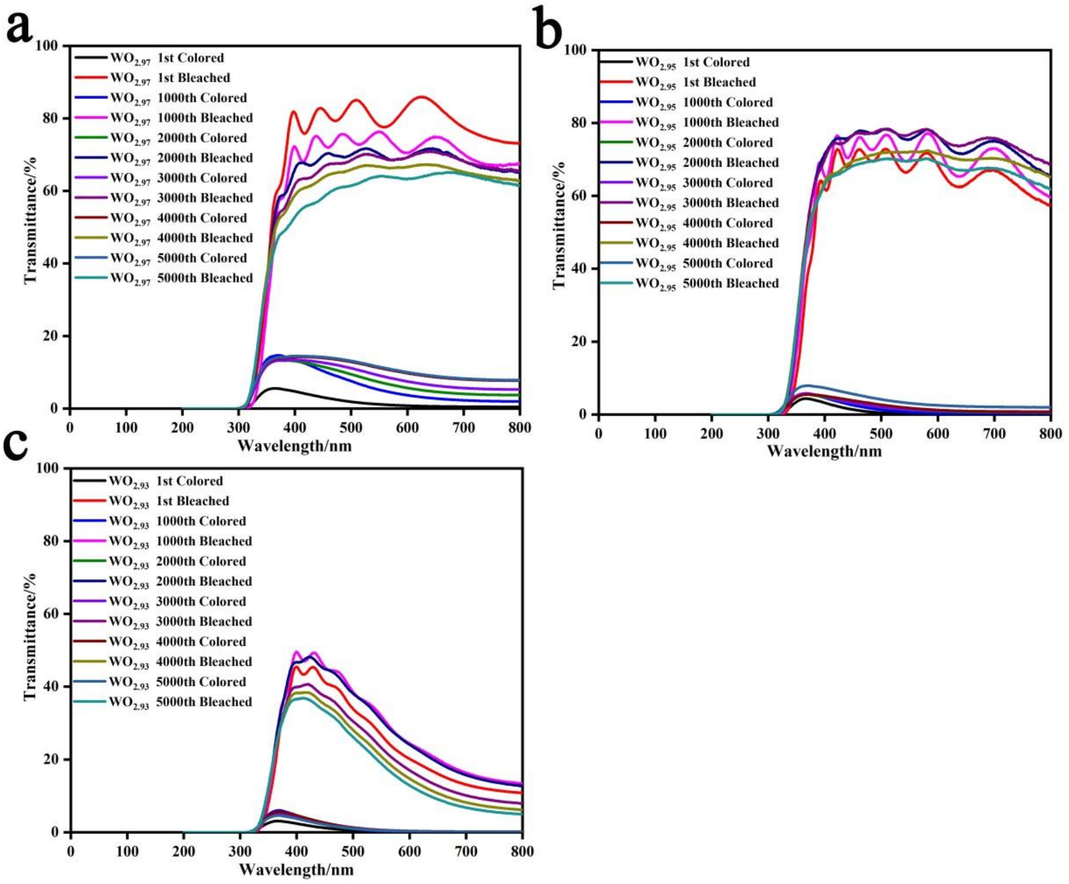

Fig.S13 Transmittance spectra evolution of tungsten oxide films with different oxygen vacancy concentrations during long-term cycling. **a.** $WO_{2.97}$, **b.** $WO_{2.95}$, **c.** $WO_{2.93}$

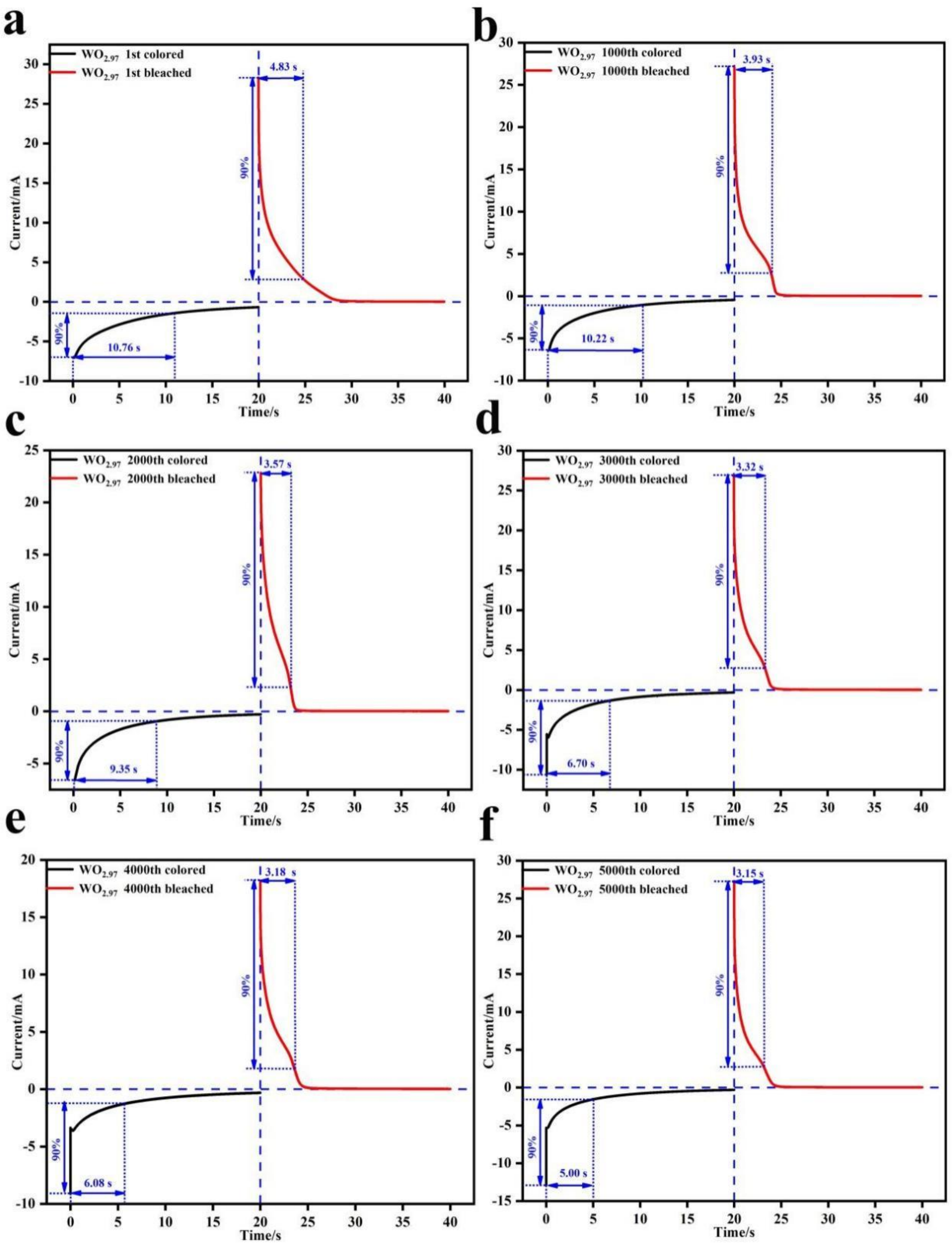

Fig.S14 Current-time curves of $WO_{2.97}$ film to test the response time during long-term cycling. **a.** 1st, **b.** 1000th , **c.** 2000th, **d.** 3000th, **e.** 4000th, **f.** 5000th

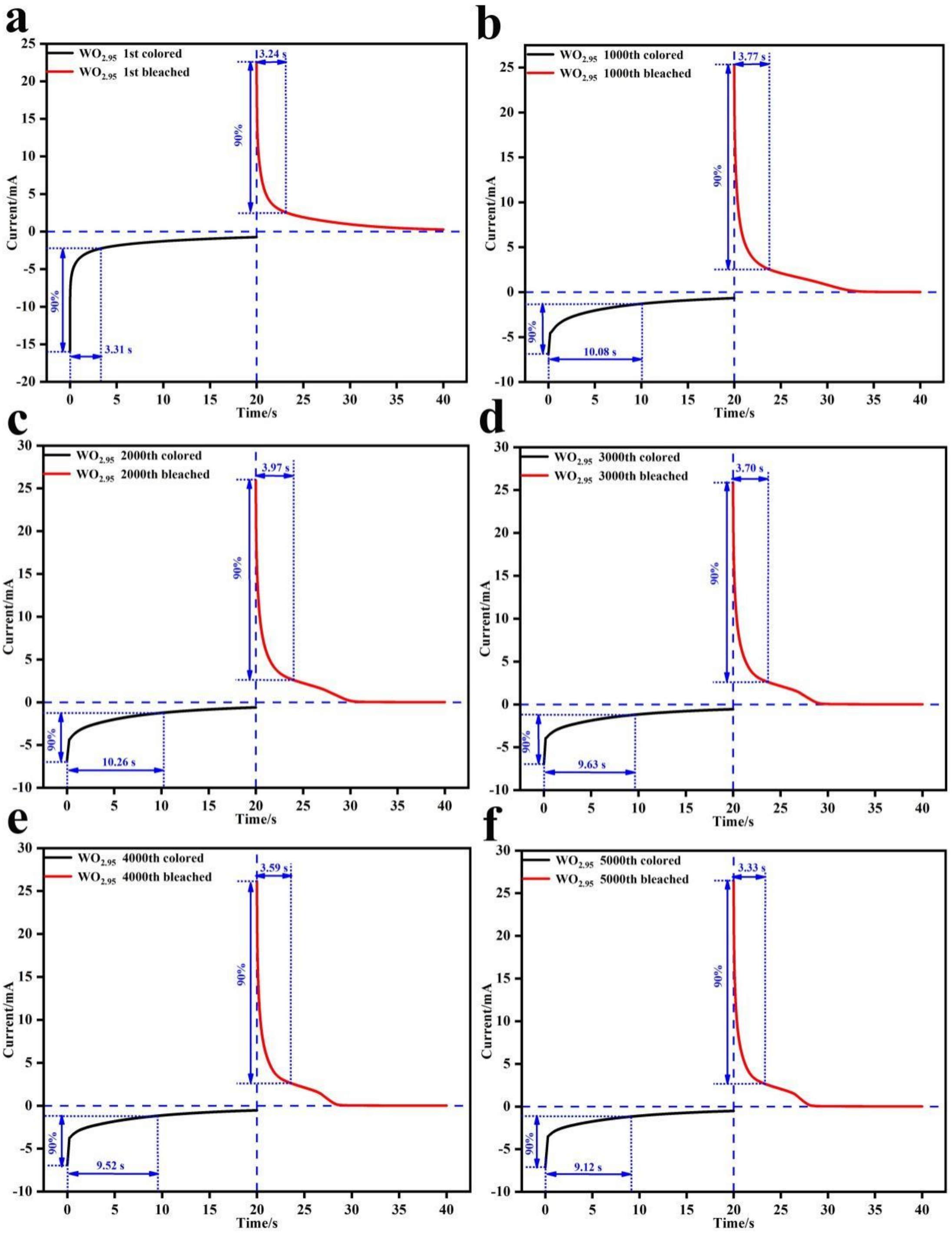

Fig.S15 Current-time curves of $WO_{2.95}$ film to test the response time during long-term cycling. **a.** 1st, **b.** 1000th , **c.** 2000th, **d.** 3000th, **e.** 4000th, **f.** 5000th

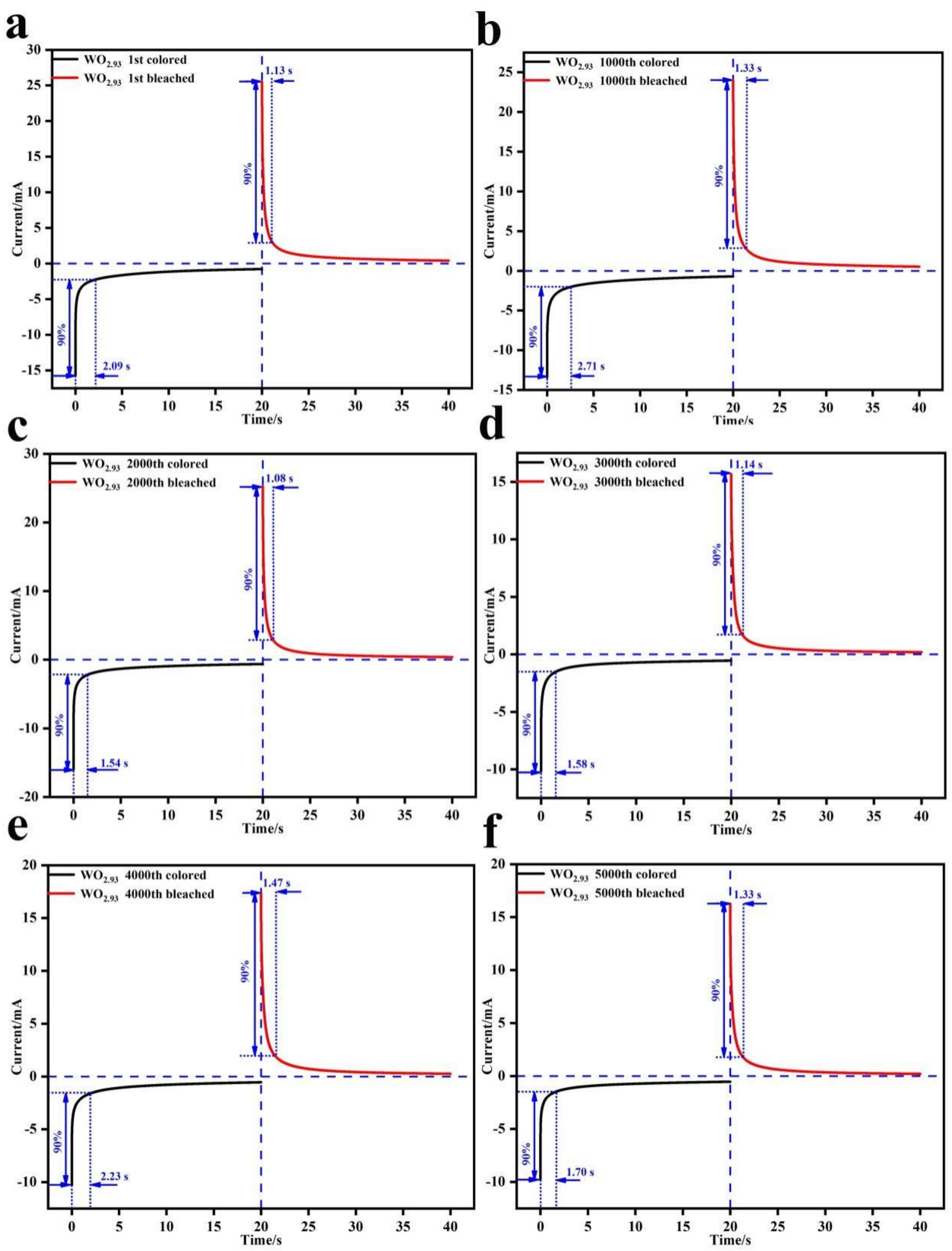

Fig.S16 Current-time curves of $WO_{2.93}$ film to test the response time during long-term cycling. **a.** 1st, **b.** 1000th , **c.** 2000th, **d.** 3000th, **e.** 4000th, **f.** 5000th

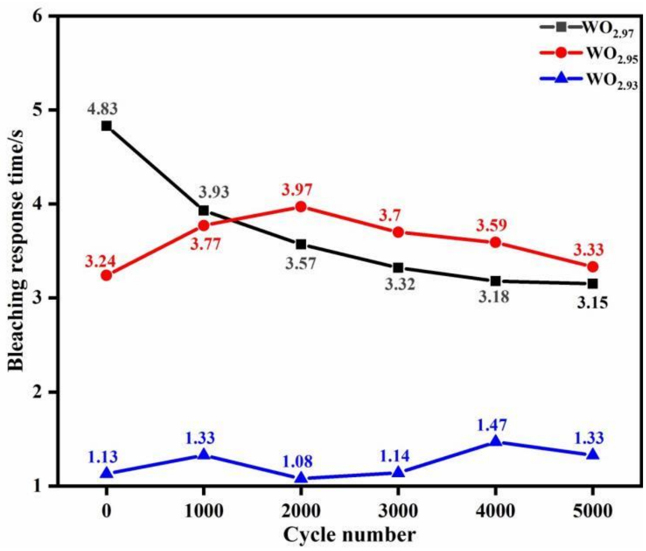

Fig.S17 Summary of the bleaching response time of tungsten oxide films with different oxygen vacancy during long-term cycling
